# Machine learning algorithms predicting bladder cancer associated with diabetes and hypertension: NHANES 2009 to 2018

**DOI:** 10.1097/MD.0000000000036587

**Published:** 2024-01-26

**Authors:** Siying Xu, Jing Huang

**Affiliations:** a Department of Urology, Wuhan Fourth Hospital, Wuhan, China.

**Keywords:** bladder cancer, diabetes, hypertension, machine learning, prediction

## Abstract

Bladder cancer is 1 of the 10 most common cancers in the world. However, the relationship between diabetes, hypertension and bladder cancer are still controversial, limited study used machine learning models to predict the development of bladder cancer. This study aimed to explore the association between diabetes, hypertension and bladder cancer, and build predictive models of bladder cancer. A total of 1789 patients from the National Health and Nutrition Examination Survey were enrolled in this study. We examined the association between diabetes, hypertension and bladder cancer using multivariate logistic regression model, after adjusting for confounding factors. Four machine learning models, including extreme gradient boosting (XGBoost), Artificial Neural Networks, Random Forest and Support Vector Machine were compared to predict for bladder cancer. Model performance was assessed by examining the area under the subject operating characteristic curve, accuracy, recall, specificity, precision, and F1 score. The mean age of bladder cancer group was older than that of the non-bladder cancer (74.4 years vs 65.6 years, *P* < .001), and men were more likely to have bladder cancer. Diabetes was associated with increased risk of bladder cancer (odds ratio = 1.24, 95%confidence interval [95%CI]: 1.17–3.02). The XGBoost model was the best algorithm for predicting bladder cancer; an accuracy and kappa value was 0.978 with 95%CI:0.976 to 0.986 and 0.01 with 95%CI:0.01 to 0.52, respectively. The sensitivity was 0.90 (95%CI:0.74–0.97) and the area under the curve was 0.78. These results suggested that diabetes is associated with risk of bladder cancer, and XGBoost model was the best algorithm to predict bladder cancer.

## 1. Introduction

Bladder cancer is 1 of the 10 most common cancers in the world, it spans a spectrum of illnesses, ranging from chronically recurrent noninvasive tumors handled noninvasively to severe or advanced-stage illness requiring multimodal and intrusive treatment.^[1,2]^ The incidence and mortality of bladder cancer are both very high, approximately 573,278 men and women are diagnosed with bladder cancer worldwide each year, more than 1.6 million living with the disease and 213,000 deaths, placing a significant burden on society.^[2]^ For men, the lifetime risk of bladder cancer is about 1.1%, whereas for women, it is about 0.27%.^[3]^ In the US, there is a documented higher occurrence of bladder cancer, accounting for more than 80,000 new cases and 17,000 deaths.^[4]^ The highest age-adjusted incidence rates of bladder cancer are found in non-Hispanic White people (23.09 [95%confidence interval (95%CI), 22.97–23.21] per 100,000 person-years), and African Americans experience worse disease-specific outcomes and higher rates of unfavorable histology.^[5,6]^

Bladder cancer has numerous risk factors, such as cigarette smoking, advanced age, gender, race, exposure to carcinogens and so on. One study found that the average age of diagnosis for bladder cancer was between 70 and 84 years, with advanced age being the biggest risk factor.^[7]^ In addition, numerous epidemiological studies have revealed a link between an elevated risk of bladder cancer and chronic disease conditions. With population aging, urbanization, and accompanying lifestyle changes, diabetic is becoming more common everywhere. Meanwhile, hypertension is becoming a bigger public health issue, and major cause of disability and the leading risk factor for death around the world. Based on International Diabetes Federation Diabetes Atlas, 9th edition, the prevalence of diabetes worldwide in 2019 is 9.3% (463 million people), and it is expected to increase to 10.2% (578 million) by 2030 and 10.9% (700 million) by 2045.^[8]^ A systematic review and meta-analysis of cohort studies indicated statistically significant relationships between diabetes (relative risk = 1.23; 95%CI: 1.16–1.31) and hypertension (relative risk = 1.07; 95%CI: 1.01–1.13) with bladder cancer.^[9]^ Bansal et al^[10]^ conducted a meta-analysis including 45 studies showed that patients with diabetes have a statistically significant (14% lower) risk of acquiring prostate cancer. Thus, the relationship between diabetes, hypertension and bladder cancer are still controversial.

Machine learning (ML), a new type of artificial intelligence algorithm, can discover data patterns and correlations in large data sets, and then use this information to determine the optimal course of action and prediction.^[11]^ At present, many ML algorithms have been widely used in medical studies, such as decision tree, random forest, extreme gradient boosting (XGBoost).^[12–14]^ We discovered that limited study has used clinical data with ML to predict the development of bladder cancer. Therefore, the aim of study was to explore the association between diabetes, hypertension and bladder cancer by using multivariate logistic regression. Additionally, we used 4 ML algorithms to predict the development of bladder cancer.

## 2. Methods

### 2.1. Study population

The dataset for this study came from the National Health and Nutrition Examination Survey (NHANES) survey conducted by the Centers for Disease Control and Prevention in the United States. The NHANES program assesses the overall health and nutrition status of adults and children, collecting information on demographic, socioeconomic, dietary, and health-related issues. In addition, laboratory tests, medical, dental and physiological assessments are carried out by extensively trained medical professionals. The NHANES used a multistage probability sample that was designed to be representative of noninstitutionalized adults in the US. In the medical condition section of the questionnaire, officials provided self-reported personal interview data. First, we used serial cross-sectional waves of NHANES from 2009 to 2018, with a total of 55,018 participants. Next, individuals were asked questions: “What kind of cancer?”. Patients with bladder cancer were selected as included subjects. After including our selected covariates and removing missing values, a total of 1789 patients were included in final analysis. Detailed statistics can be accessible at https://www.cdc.gov/nchs/nhanes/.

### 2.2. Diagnostic criteria

The diagnostic criteria for hypertension were: a resting systolic blood pressure ≥ 140mmHg and/or a diastolic blood pressure ≥ 90 mm Hg. Diabetes was defined a documented history in the medical record. Body mass index (BMI) was calculated as weight in kilograms divided by height in meters squared (kg/m^2^). Three BMI classifications were used < 25, 25 to 30, and ≥ 30 kg/m^2^, reflecting underweight or normal weight, overweight, and obesity, respectively. Sleep quality is measured based on the length of sleep at night, and <7 hours is considered poor.

### 2.3. Covariates

Other covariate data were also collected. Age was calculated from the interview date to the birth date. Gender was self-reported as male or female. Race was categorized to 5 group: Mexican American, Other Hispanic, Non-Hispanic White, Non-Hispanic Black and Other Race. Education was grouped according to the following categories: less than high school (<grade 9), 9 to 11th grade, high school, some college and college graduate or above. Marital Status was categorized as married, widowed, divorced, separated, never married, living with partner, and refused. PIR refers to family poverty income ratio, where a PIR value <1 indicates an income below the poverty level and a PIR value larger than 1 indicates an income over the poverty threshold. Both smoking and alcohol drinking was separated into 2 categories: yes or no. Blood pressure was measured using a mercury sphygmomanometer, 3 consecutively reading of systolic blood pressure and diastolic blood pressure were taken 5min intervals. The mean of the 3 readings was calculated in the analysis.

### 2.4. Statistical analysis

Continuous variables were displayed as mean (standard deviation). Differences in continuous variables between groups were tested with Student *t* test. Categorical variables were provided as percentages, and differences in categoric variables between groups were assessed by *χ*^2^ tests. Multivariate logistic regression model was performed to estimate the association between hypertension, diabetes and bladder cancer. Covariates were adjusted for the following 4 models: Model 1 = unadjusted; Model 2 = hypertension/diabetes; Model 3 = Model 2 + sex, age (continuous), education, race and BMI; Model 4 = Model 3 + smoking, alcohol drinking, and sleep quality. In order to better predict bladder cancer, we first carried out random forest interpolation on all data to ensure sufficient sample size and variables. After interpolation, the sample size for ML reached 36,149, with sufficient data to achieve the best prediction effect. We selected 4 highly recognized ML algorithms: XGBoost, Artificial Neural Networks, Random Forest, and Support Vector Machine. In order to achieve the best predictive effect of ML, we applied the random forest algorithm to interpolate missing values. The comparison between the 4 algorithms was based on the accuracy of the predictions and the Kappa value. As shown in Supplementary Figure S1, http://links.lww.com/MD/L35, we drew the density plot after interpolating the severely missing drinking data, and found that the inserted value was consistent with the true value, and the interpolation effect was good. The ML model performance was assessed using the accuracy, precision, sensitivity, specificity, and area under the receiving operating characteristic curve (AUC). All the analyses were conducted in R software 4.1.2 (The R Foundation for Statistical Computing, USA). Two-sided *P* < .05 was considered statistically significant.

### 2.5. Ethical approval

The institutional review board of the National Center for Health Statistics, a division of the Centers for Disease Control and Prevention, gave its approval to the NHANES protocols. Before beginning the study, written informed consent was obtained from each participant.

## 3. Results

### 3.1. The clinical characteristics

The basic characteristics of participants grouped according to whether they had bladder cancer are presented in Table 1. The mean age of bladder cancer group was older than that of the non-bladder cancer (74.4 years vs 65.6 years, *P* = .0009). Gender was significantly associated with bladder cancer; men were more likely to have bladder cancer (*P* = .011). Other variables were not statistically different between the 2 groups, including PIR, BMI, race, education, marital status, alcohol, smoking, hypertension, diabetes and sleep quality (*P* > .05).

**Table 1:** The basic characteristics of participants grouped according to whether they had bladder cancer.

Characteristics	Non-bladder cancer (N = 1752)	Bladder cancer (N = 37)	*F* or *χ*^2^
Age (mean, SD)	65.6 ± 13.7	74.4 ± 7.4	0.0009*
Gender (n, %)			0.011*
Male	835 (47.7%)	26 (70.3%)	
Female	917 (52.3%)	11 (29.7%)	
PIR	2.68 (1.61)	2.38 (1.59)	0.296
BMI (n, %)			0.285
Normal	410 (25.8%)	8 (22.2%)	
Overweight	631 (39.6%)	11 (30.6%)	
Obesity	551 (34.6%)	17 (47.2%)	
Race (n, %)			0.392
Mexican American	111 (6.34%)	2 (5.41%)	
Other Hispanic	118 (6.74%)	0 (0.00%)	
Non-Hispanic White	1135 (64.8%)	24 (64.9%)	
Non-Hispanic Black	269 (15.4%)	7 (18.9%)	
Other Race	119 (6.79%)	4 (10.8%)	
Education (n, %)			0.805
<9th grade	166 (9.5%)	4 (10.8%)	
9–11th grade	212 (12.1%)	6 (16.2%)	
High school	380 (21.7%)	7 (18.9%)	
Some college	542 (30.9%)	9 (24.3%)	
College graduate or above	452 (25.8%)	11 (29.7%)	
Marital status (n, %)			0.596
Married	916 (52.3%)	22 (59.5%)	
Widowed	326 (18.6%)	10 (27.0%)	
Divorced	253 (14.4%)	3 (8.11%)	
Separated	60 (3.4%)	0 (0.0%)	
Never married	128 (7.3%)	1 (2.7%)	
Living with partner	62 (3.54%)	1 (2.7%)	
Refused	7 (0.40%)	0 (0.0%)	
Alcohol			0.96
Yes	33 (15.3%)	0 (0.0%)	
No	183 (84.7%)	3 (100%)	
Smoking			0.096
Yes	966 (55.1%)	26 (70.3%)	
No	786 (44.9%)	11 (29.7%)	
Hypertension			0.97
Yes	47 (3.7%)	1 (3.1%)	
No	1234 (96.3%)	31 (96.9%)	
Diabetes			0.356
Yes	380 (21.7%)	11 (29.7%)	
No	1299 (74.1%)	24 (64.9%)	
Borderline	73 (4.16%)	2 (5.41%)	
Sleep quality			0.784
Poor	1212 (69.5%)	27 (73.0%)	
Good	532 (30.5%)	10 (27.0%)	

* *P* < .05.

BMI = body mass index, PIR = ratio of family income to poverty, SD = standard deviation.

### 3.2. Association between diabetes, hypertension and bladder cancer

Using Logistic regression models adjusted with different confounding factors, we explored the associations between diabetes, hypertension and bladder cancer (Table 2). In fully adjusted model, diabetes was significantly associated with bladder cancer (odds ratio = 1.24, 95%CI: 1.17–3.02). There was no statistical significance between hypertension and bladder cancer. Hypertension may not be a risk factor for bladder cancer.

**Table 2:** Logistic regression analysis for diabetes, hypertension and bladder cancer (odd ratios [OR] and 95%CI).

	Model 1	Model 2	Model 3	Model 4
Diabetes				
No	1.00	1.00	1.00	1.00
Borderline	1.35 (0.17, 10.51)	1.35 (0.16, 11.18)	1.79 (0.18, 1.87)	1.66 (0.18, 5.75)
Yes	1.27 (0.41, 3.99)	1.16 (0.31, 3.64)	1.03 (0.18, 3.00)	1.24 (1.17, 3.02)
Hypertension				
No	1.00	1.00	1.00	1.00
Yes	5.09 (0.62,41.71)	5.04 (0.61,41.45)	10.46 (0.74,148.44)	11.11 (0.73,168.93)

Model 1: Unadjusted; Model 2 = Adjusted for hypertension (when studying the relationship between diabetes and bladder cancer); adjusted for diabetes (when studying the relationship between hypertension and bladder cancer); Model 3: Adjusted for hypertension/diabetes, sex, age (continuous), education, race and BMI; Model 4 = Adjusted for hypertension/diabetes, sex, age (continuous), education, race and BMI, smoking, alcohol drinking, and sleep quality.

95%CI = 95%confidence interval, BMI = body mass index.

### 3.3. ML algorithms to predict bladder cancer

**Figure 1.**
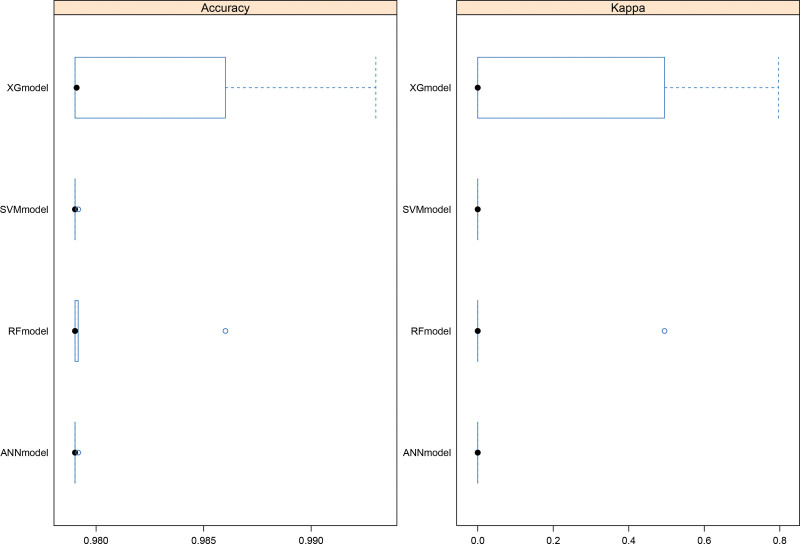
Comparison of accuracy and kappa value among 4 machine learning algorithms.

**Figure 2.**
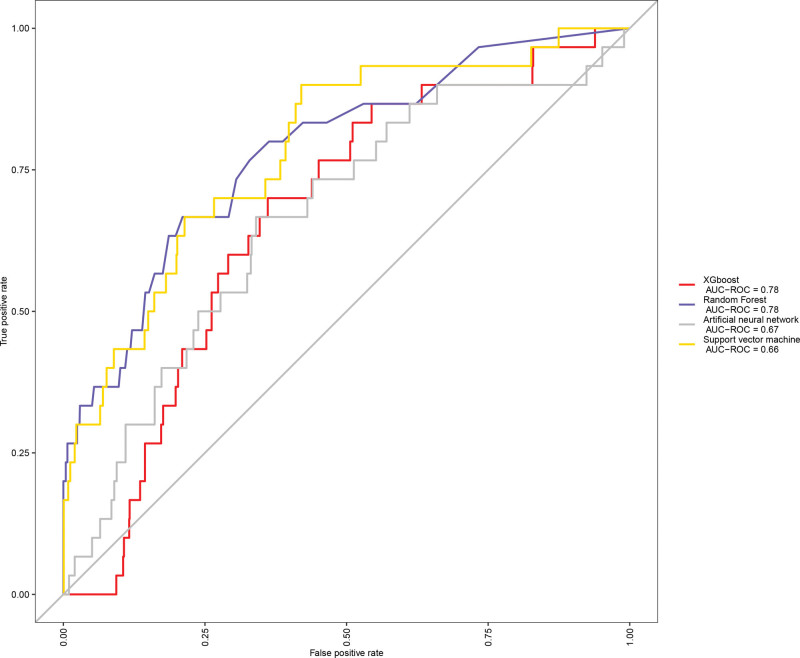
Receiver operating characteristic (ROC) curve of diabetes, hypertension and bladder cancer by using 4 machine learning algorithms.

Figure 1 compares the fit plots after placing all covariables into 4 ML algorithms. We concluded that the XGBoost model fit the best (accuracy = 0.978 with 95%CI:0.976–0.986; kappa value = 0.01 with 95%CI: 0.01–0.52). We also produced receiver operating characteristic curve to evaluate the prediction effect of these 4 ML algorithms (Fig. 2). The AUC values were calculated for 4 models (XGBoost AUC = 0.78; Random Forest AUC = 0.78; Artificial Neural Networks AUC = 0.67; Support Vector Machine AUC = 0.66, respectively), which demonstrated that XGBoost predicted well. In order to further check the evaluation results, we also show the evaluation parameters of 4 ML algorithms in Table 3. XGBoost algorithm has the best prediction effect on bladder cancer based on clinical data.

**Table 3:** Detailed comparison among 4 machine learning algorithms for ROC curves.

Item	XGBoost	Random forest	ANN	SVM
Sensitivity	0.90 (95%CI:0.74–0.97)	0.667 (95%C:0.49–0.81)	0.70 (95%CI: 0.52–0.83)	0.667 (95%CI:0.49–0.81)
Specificity	0.58 (95%CI:0.55–0.61)	0.79 (95%CI:0.77–0.81)	0.639 (95%CI: 0.61–0.66)	0.66 (95%CI:0.63–0.68)
Prediction	0.044 (95%CI:0.03–0.06)	0.063 (95%CI:0.04–0.10)	0.04 (95%CI:0.03–0.06)	0.04 (95%CI:0.03–0.06)
Negative prediction	0.996 (95%CI:0.99–1.00)	0.991 (95%CI: 0.98–1.00)	0.99 (95%CI:0.98–0.99)	0.989 (95%CI:0.98–0.99)
AUC	0.78 (95%CI:0.68–0.88)	0.78 (95%CI: 0.68–0.88)	0.67 (95%CI:0.56–0.78)	0.66 (95%CI:0.55–0.77)
Matthews correlation coefficient	0.139	0.158	0.101	0.098
False positive rate	0.42	0.21	0.361	0.34
F1 value	0.084	0.116	0.075	0.076
True positive	27	20	21	20
False positive	589	295	506	477
True negative	813	1107	896	925
False negative	3	10	9	10

95%CI = 95%confidence interval, ANN = Artificial Neural Networks, AUC = area under the subject operating characteristic curve, ROC = receiver operating characteristic, SVM = Support Vector Machine, XGBoost = extreme gradient boosting.

## 4. Discussion

In this study, we analyzed 1789 patients from NHANES and found that advanced age and gender were important risk factors for bladder cancer. Results indicated that men were more likely to have bladder cancer, which was consistent with previous study.^[15]^ Men are 3 to 4 times more likely to be diagnosed with bladder cancer than women, a fact that is generally related to exposures and lifestyle.^[16]^ At the epidemiologic level, large cohort studies have shed light on the potential contribution of sex steroids to bladder cancer, for instance, postmenopausal women have a larger chance of developing bladder cancer than do premenopausal women.^[17]^ At the molecular level, the underlying mechanism responsible for the differences in bladder cancer incidence may be sex differences in carcinogenic cell metabolism. In the Nurses’ Health Study (NHS) and NHS II, younger age at menopause (≤45 years) was associated with an increased risk of bladder cancer (incidence risk ratios: 1.41, 95%CI: 1.11–1.81), relative to those with menopause beginning at age 50 + years.^[17]^ Numerous clinical studies have found that the median age of diagnosis for bladder cancer is greater than for other major tumors.^[18]^ In addition to changes in the gut and urinary tract microbiota and elevated indicators of chronic inflammation, aging is related with an increased incidence, morbidity, and death of bladder cancer. Age-related changes in a variety of microbiomes may be the cause of the rising incidence and mortality of bladder cancer in the elderly. These changes may generate systemic metabolic modifications that impact immunological dysregulation.^[19]^

Herein, we also explored the association between diabetes, hypertension and bladder cancer. Diabetes has been suggested as an important risk factor for bladder cancer; however, conflicting results have emerged. Choi et al^[20]^ found that diabetes was associated with an increased risk of bladder cancer (hazard ratio = 1.23, 95%CI:1.17–1.28), which was consistent with our study. A nationally representative study from the Korean National Health Insurance System also illustrated there was a strongly positive association between diabetes and bladder cancer.^[21]^ In a case-control study, diabetes was linked to an elevated risk of bladder cancer (adjusted odds ratio: 2.2, 95%CI, 1.3–3.8).^[22]^ However, a recent meta-analysis showed that impaired fasting glucose was not associated with the risk of bladder cancer.^[23]^ The relationship between diabetes and bladder cancer has not been fully established, and even an inverse association has been observed. The biochemical connections between diabetes and bladder cancer are as follows: aberrant insulin/insulin-like growth factor axis activity, inflammatory cytokines, hyperglycemia, faulty sex hormone biosynthesis, and elevated oxidative stress that causes DNA damage.^[24,25]^ Urinary tract infections, which are common in diabetes patients, are another factor in the development of bladder cancer, and bacterial cell components can cause cellular growth and inflammation, potentially accelerating the development of cancer.^[26]^ Therefore, early prevention and treatment in patients with diabetes could effectively reduce the occurrence of bladder cancer.

Findings from this study found that there was no significant association between hypertension and bladder cancer, which is inconsistent with the conclusions of some studies. Epidemiologic evidence involving 79,236 propensity score-matched individuals found a positive association between hypertension and subsequent bladder cancer development.^[27]^ Untreated hypertension was associated with a reduced risk of bladder cancer.^[28,29]^ The following justifies the link between high blood pressure and bladder cancer: hypertension is a metabolic syndrome component that has been linked to the development of cancer in the future. It is evident that there is conflicting evidence about the link between high blood pressure and bladder cancer. Over 116 million adults in the US and over 1 billion people globally suffer from hypertension, which is a major cause of CVD morbidity and mortality. The weighted prevalence of hypertension was 46.7%, with 80.1% of cases being uncontrolled.^[30]^ Given that high blood pressure and bladder cancer are both serious health problems and the question of the link between the 2 remains unresolved, there is an urgent need to explore whether people with high blood pressure are at higher risk of developing bladder cancer so that appropriate prevention and treatment measures can be taken early.

ML is increasingly being used in clinical oncology to diagnose cancer, predict patient outcomes, and inform treatment plans. ML has many benefits, such as the ability to recognize trends and patterns quickly, the lack of human interaction, ongoing progress, and a broad range of applications, which can significantly increase the accuracy of prognosis and diagnosis when applied to clinical data.^[31]^ In this study, by using multivariate logistic regression to determine the risk factors, then we adopted 4 ML models to predict bladder cancer. It could be seen from the comparison plot that XGBoost outperformed the other algorithms, the AUC was 0.78. Kouznetsova et al^[32]^ used 2 modeling techniques: multilayer perceptron and stochastic gradient descent with logistic regression loss function to locate bladder cancer patients, with an accuracy of 82.54%. XGBoost we performed in this study provided a better sensitivity and specificity.

However, it has some limitations. Firstly, the cross-sectional study design from NHANES precluded establishing causality between diabetes, hypertension and bladder cancer. Second, in an observational study, residual confounders could not be completely ruled out, for example, diet, stress, physical activity and genetic factors are known confounders for bladder cancer, these variables were not accounted for in the analysis. Further studies are needed to clarify the mechanism in the relationship between diabetes, hypertension and bladder cancer.

## 5. Conclusions

Bladder cancer is the most common malignancy of the urinary tract. Our analysis further confirmed the significant effects of diabetes on bladder cancer. The clinical significance of ML in identifying and predicting disease frequency and progression as well as other risk factors for blader cancer can be defined by additional study.

## Author contributions

**Conceptualization:** Jing Huang.

**Formal analysis:** Siying Xu, Jing Huang.

**Writing – original draft:** Siying Xu, Jing Huang.

**Writing – review & editing:** Jing Huang.

## Supplementary Material


