# Supplementary figures and images for: Machine learning algorithms predicting bladder cancer associated with diabetes and hypertension: NHANES 2009 to 2018
Source: Medicine (Baltimore). 2024 Jan 26;103(4):e36587. doi: 10.1097/MD.0000000000036587 (PMC10817101; doi:10.1097/MD.0000000000036587)

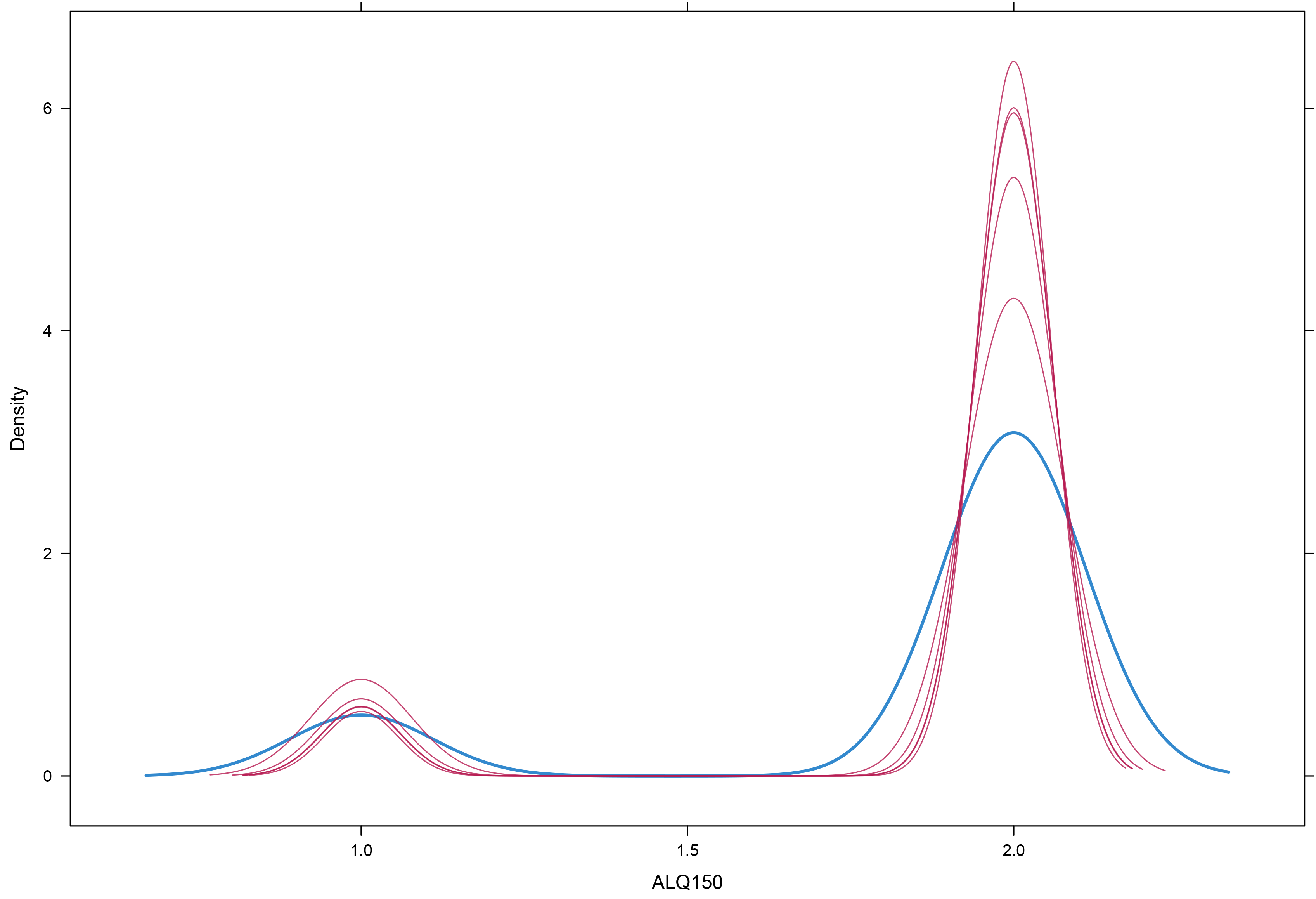

Supplement: Supplementary file 1 [file medi-103-e36587-s001.tif]
